# Associations of pro-inflammatory factors and IL-10 levels with degree of suicide risk in adolescents with depression

**DOI:** 10.3389/fpsyt.2024.1491555

**Published:** 2025-01-15

**Authors:** Wenyuan Liu, Hongyu Zheng, Xiaofei Wen, Longxing Liu, Yue Yang, Hui Zhong

**Affiliations:** ^1^ School of Mental Health and Psychological Sciences, Anhui Medical University, Hefei, China; ^2^ Department of Child and Adolescents Psychology, Anhui Mental Health Center, Hefei, China; ^3^ Department of Child and Adolescents Psychology, Affiliated Psychological Hospital of Anhui Medical University, Hefei, China; ^4^ Department of Child and Adolescents Psychology, Hefei Fourth People’s Hospital, Anhui, China

**Keywords:** suicide risk, depression, adolescence, pro-inflammatory state, anti-inflammatory factors, IL-10

## Abstract

**Background:**

Depression and suicidal behavior are associated with pro-inflammatory status in adults. However, differences in inflammatory levels among adolescents with depression at different suicide risk levels are unclear, and the connection between anti-inflammatory factors, which serve as vital for the immune system, and suicide needs to be explored.

**Methods:**

This study recruited 111 adolescent patients with depression aged 13-18 and 23 healthy controls. Patients were divided into three subgroups according to suicidal ideation within the past week and history of suicide attempts. Severity of depression, suicidal ideation, and suicide risk were assessed using the Hamilton Depression Scale-17 (HAMD-17) and the Chinese version of the Beck Suicide Ideation Scale (BSI-CV). Plasma levels of IL-6, TNF-α, IFN-γ, IL-1β, and IL-10 in all participants were measured.

**Results:**

Plasma levels of IL-6, TNF-α, IFN-γ, and IL-10 differed between the suicide risk subgroups, and the differences remained significant after controlling for severity of depressive symptoms using covariance analysis. Pairwise comparison indicated that plasma levels of these four cytokines in the high suicide risk group were higher than those in the low suicide risk group (all p<0.05), among which the level of IL-10 was significantly higher than that in the medium and low risk groups. IL-10 was positively correlated with the total score of the HAMD-17, BSI-CV, and suicidal ideation; the other four cytokines were also somewhat correlated with suicidal ideation (all p<0.05). IL-10 correlated positively with these four pro-inflammatory factors. Multiple linear regression analysis showed that IL-10 levels significant were associated with BSI-CV (β = 0.270, t = 2.897, p = 0.005) and HAMA-17 (β = 0.285, t = 3.041, p = 0.003) total scores. In binary logistic regression, after controlling for depressive symptoms, gender, age, BMI, and duration of illness: IL-10 level remained a risk factor for suicidal behavior (OR = 3.224, 95% CI 1.571-6.619 p = 0.001).

**Conclusion:**

Adolescents with different suicide risk levels differed in plasma levels of pro-inflammatory factors and the anti-inflammatory factor IL-10. These differences were independent of depressive symptoms; high IL-10 levels may be a risk factor for suicidal behavior in depressed patients. Further research is needed to explore the relationship between anti-inflammatory factors and suicide.

## 1 Introduction

In recent years, the problem of suicide has gradually attracted widespread attention internationally and from the International Health Organization, specifically, with data indicating that globally about 800,000 people die of suicide each year (1). Suicide has become one of the most important causes of death among adolescents, accounting for about 8.5% of all deaths among people aged 15-29 years (2). Mental disorders, especially major depressive disorder (MDD), have a dramatic impact on suicide rates, accounting for approximately 30% of suicides (3), while increasing the probability of suicide more than three-fold (1). MDD is a predictor of adolescent suicidal behavior (4), and suicide thoughts are far more common in teenagers who are depressed than their general peers (5). Suicidal behavior in adolescents with major depression causes great suffering to the patients themselves and their families, as well as an incalculable economic burden. Therefore, the search for possible clinical interventions has become urgent. However, neurophysiological mechanisms underlying suicidal behavior in adolescents with major depression remain unexplored (6).

Inflammatory factors, a biomarker that potentially influences depression and suicide risk, affect susceptibility to neuropsychiatric disorders and suicide by influencing the synthesis and metabolic pathways of monoamine neurotransmitters. Previous studies noted higher blood levels of inflammatory biomarkers (including IL-6, IL-1β, TNF-α, and CRP) in people with mental illness who have suicidal attempts in comparison to healthy controls or non-suicidal people with mental illness (7–10). High plasma levels of cytokines in depressed patients were likewise correspondingly associated with suicidal ideation (11, 12). The relationship between suicide, MDD, and inflammation is complex. Depression involves a bidirectional disturbance related to inflammation, and numerous studies confirming their reciprocal reinforcement (13). However, a small number of studies have reported a lack of substantial association between depression and elevated cytokine levels, and levels of inflammatory factors may be negatively correlated with depressive symptoms in some patients (14–17). The roles of suicide and inflammation also tend to be reciprocal; on the one hand, inflammation may induce suicidal ideation, with one study showing an increase in suicidal ideation in patients with hepatitis C and multiple sclerosis who received pro-inflammatory cytokine therapy compared to other treatments (18, 19). On the other hand, suicidal ideation may enhance inflammatory responses. For instance, suicide-related threat perceptions may activate certain stress responses, including inflammatory responses (20).

Cytokines are categorized into anti-inflammatory and pro-inflammatory cytokines based on the role they play in the inflammatory response. Under normal physiological conditions, pro-inflammatory and anti-inflammatory cytokines tend to remain in a relatively balanced state, and anti-inflammatory factors are usually accompanied by changes in pro-inflammatory factors in cases of immune dysfunction. However, existing studies on inflammation and suicide have mostly studied pro-inflammatory cytokines as biomarkers, and the role of anti-inflammatory factors has been the subject of very little research. IL-10, one of the most important anti-inflammatory cytokines, is involved in various disease mechanisms and has been widely studied. Previous studies have shown that depressive symptoms exhibit a strong correlation with elevated levels of IL-10 *in vivo* (21–23). Therefore, further understanding of the role of IL-10 in depression suicide is required.

Most previous studies on depression suicide and changes in inflammation levels have focused on adults, while relatively few studies have been undertaken on adolescents.; as cytokine levels tend to change with age, differences may exist between adolescents and adults in the underlying pathophysiology of suicide. Additionally, the majority of studies have focused only on differences in inflammatory factors between depressed patients in general and those with suicidal ideation/behavior, with less attention paid to differences between the two. Therefore, the present study sought to examine variations in levels of pro-inflammatory and anti-inflammatory factors among adolescent depressed patients with three different suicide risk levels. We chose four pro-inflammatory cytokines whose changes in levels have previously been shown to strongly correlate with suicidal ideation or behavior in adults and these cytokines show abnormalities in the cerebrospinal fluid of suicide victims: IL-6 (24–28), IL-1β (29–33), TNF-α (34–38), IFN-γ (39–42). On this basis, we further explored the relationship between the anti-inflammatory factor IL-10 and suicide risk. We also explored correlations between the severity of depressive symptoms, suicidal ideation, and suicide risk with inflammatory factors, as well as searching for risk factors affecting suicidal behavior in adolescent with major depression.

## 2 Methods

### 2.1 Sample

This study was ethically reviewed by the Fourth People’s Hospital of Hefei City (review number: HSY-IRB-PJ-XJJ-ZH002). We recruited 111 adolescents with major depressive disorders (MDD; n = 111) who attended the Department of Child and Adolescent in the Fourth People’s Hospital of Hefei City from June 2019 to April 2021. Inclusion criteria: (1) met diagnostic criteria for major depressive disorders in DSM-5; (2) aged 13–18 years; (3) No medication has been used in the last two weeks. Exclusion criteria: (1) comorbidity with neurological disease, serious physical illness, psychoactive substance abuse, intellectual disability; (2) comorbid other psychiatric disorders; (3) comorbid infectious diseases, or diseases that may affect inflammation level. Twenty-three healthy controls (HC; n = 23) comprising Hefei City general secondary school students matching the age and years of education of the depressive disorder group were simultaneously recruited. Inclusion criteria: (1) aged 13-18 years; (2) Hamilton Depression Scale score less than 7; (3) no history of mental illness in the person and for three generations of both lines. Exclusion criteria were the same as those for the depressive disorder group. All participants and their families gave informed consent and signed a paper version of the informed consent form prior to the study.

### 2.2 Clinical assessment

Clinical Assessment: (1) General Conditions: This included participants’ gender, age, body mass index (BMI), years of education, family history, disease duration, and whether suicidal behavior had occurred, etc. (2) Scale Assessments: Participants were scored by two attending physicians who had undergone consistency training, using the Hamilton Depression Scale-17 items (HAMD-17) to assess the severity of depressive symptoms. This scale has 17 items; higher scores indicate more severe depressive symptoms. The Beck Suicide Ideation Scale Chinese Version (BSI-CV) was used to assess the intensity, duration, and specific characteristics of the patient’s plans and wishes for suicide. This scale consists of two factors: suicidal ideation (first 5 items) and suicidal tendency (last 14 items), with a total of 19 items using a three-level scoring system, each item scored from 0 to 2, and the total score ranged from 0 to 38. Participants were only considered to have suicidal ideation if their responses to item 4 (active suicidal ideation) or item 5 (passive suicidal ideation) on the scale were not “none.” They then proceeded to answer the subsequent questions 6 to 19. If no suicidal ideation existed, the total score was the sum of the first five items. The higher the score of the first five items, the higher the intensity of suicidal ideation; the higher the total score, the higher the risk of suicide. Patients were divided into three subgroups on the base of history of suicide attempts and items 4-5 of the Beck Suicidal Ideation Inventory: (1) High suicide risk group (HR: n = 45): at least one of the responses to items 4-5 of the Beck Suicidal Ideation Inventory (active and passive suicide ideation) was “weak” or “moderate to strong,” and there was a previous history of suicide attempts; (2) Medium Suicide Risk Group (MR; n = 44): at least one of the responses to items 4-5 of the Beck Suicidal Ideation Inventory was “weak” or “moderate to strong” but no history of suicide attempts; (3) Low suicide risk group (LR: n = 22): “none” on items 4-5 of the Beck Suicidal Ideation Scale and no history of suicide attempts.

### 2.3 Blood sample collection and cytokine analysis

Both clinical assessment and blood collection were performed on the same morning. Participants fasted for more than 8 hours before the blood draw, and 5 ml of blood was withdrawn from the elbow vein in EDTA anticoagulant tubes. The blood was centrifuged (2000 r/min, 20 min) within one hour, and the supernatant was stored in a refrigerator at -80°C immediately afterward. Then an ultrasensitive multifactor electrochemiluminescence analyzer (Model SQ120) (MESOTM Quick-Plex SQ120) was uniformly used to determine the plasma IL-6, TNF-α, IFN-γ, IL-10, and IL-1β concentrations through the steps of room-temperature incubation, dilution and oscillation, plate washing, dilution and oscillation, plate washing, addition of antibodies, plate washing, and addition of plate reader solution. concentration in plasma. The assay sensitivities (min, max) are (0.0619pg/mL, 1870 pg/mL) for IL-6, (0.160pg/mL, 3460pg/mL) for TNF-α, (0.254pg/mL,28500pg/mL) for INF-γ, (0.0126pg/mL, 3740pg/mL) for IL-10 and (0.0118pg/mL, 4040pg/mL) for IL-1β. The average intra-assay CV was 3.46% for IL-6, 5.66% for TNF-α, 4.25% for IFN-γ, 4.35% for IL-10 and 2.98% for IL-1β. In addition, the inter-assay CVs reported by the manufacturer were less than 10% for all five cytokines.

### 2.4 Statistical analysis

Statistical analysis was performed using SPSS 25.0. Shapiro-Wilk was used for all continuous variables to test normality. Measurement data that conformed to a normal distribution were expressed as mean (M) ± standard deviation (SD), and comparisons between groups were made using independent sample t-tests or one-way ANOVA. For variables that did not follow a normal distribution, they were expressed as median and interquartile range expressed as M (Q1, Q3), and comparisons between groups were made using non-parametric tests such as Mann-Whitney U or Kruskal-Wallis H tests. *Post hoc* pairwise comparisons were adjusted for using the Bonferroni method. Categorical data were expressed as frequencies and rates, and intergroup comparisons were made using the chi-square test. Spearman’s correlation analysis was used to investigate the correlation between plasma cytokine concentrations in patients with depression and HAMD-17 total score, and BSI-CV total score and suicidal ideation score. Analysis of covariance was used to exclude confounding factors, regression analysis was used to explore the risk factors affecting suicidality and depressive symptoms. Prior to ANCOVA and regression analyses, plasma cytokine concentrations were subjected to a natural logarithmic transformation to attenuate skewness to converge to a normal distribution. Multiple linear regression was used to analyze the effects of gender, age, BMI, disease duration, and inflammatory factor levels on HAMD-17 and BSI-CV total scores in depressed patients. Finally, to explore the effects of cytokine levels on suicide risk, we used a binary logistic regression model to analyze the independent risk factors affecting suicidal behavior, with previous suicidal acts as the dependent variable and sociodemographic data, severity of depressive symptoms, and cytokines that demonstrated statistical significance in the univariate regression model as independent variables. The diagnostic predictive efficacy was further analyzed using ROC curve analysis. All tests were two-tailed, and a p-value<0.05 was considered statistically significant.

## 3 Results

### 3.1 Sample characteristics

A total of 111 adolescents with depression and 23 healthy controls were recruited; sociodemographic and clinical case data are shown in **Table 1**. No significant differences were found between the suicidal behavior subgroups and HCs in terms of gender, age, BMI, and years of education (x^2^ = 3.703, p=0.295 for gender; F=1.811, p=0.148 for age; F=2.409, p=0.070 for BMI; F=0.696, p=0.556 for years of education); illness duration did not significantly differ between the suicidal behavior subgroups (H=0.102, p=0.950). As expected, severity of depressive symptoms and level of suicidal risk were higher in the MR and HR groups than in the low-risk group, that is, HAMD-17 scores differed between the suicidal behavior subgroups (F=5.658, p=0.005). There were significant differences in the BSI-CV total scores, and the suicidal ideation scores (H=49.330, p<0.001 for BSI-CV total scores; H=41.700, p<0.001 for suicidal ideation scores). *Post hoc* two-by-two comparisons showed that all four scores were higher in the MR and HR groups compared to the LR group, while there was no significant difference in scores between the MR and HR groups.

**Table 1:** Sociodemographic, clinical, and inflammatory characteristics stratified by suicide risk.

Characteristics	HC (n=23)	HR (n=45)	MR (n=44)	LR (n=22)	F/H/x²	P	P#
Age	15.83 ± 1.53	14.91 ± 1.54	15.14 ± 1.53	15.18 ± 1.59	1.811	0.148	
BMI (kg/m^2^)	21.50 ± 3.35	23.85 ± 6.96	21.45 ± 4.52	21.04 ± 2.40	2.409	0.070	
Years of education	9.87 ± 1.58	9.38 ± 1.50	9.61 ± 1.50	9.77 ± 1.27	0.696	0.556	
Gender (M/F)	7/16	11/34	11/33	10/12	3.703	0.295	
Disease Duration	N/A	12 (6, 24)	12 (6, 24)	12 (6, 24)	0.102	0.950	
Family history	N/A	5/40	8/36	1/21	2.629	0.269	
HAMD-17 scores	N/A	20.18 ± 7.30	21.02 ± 7.21	14.77± 7.78	5.658	0.005^*^	HR, MR>LR
BSI-CV total scores	N/A	19 (9, 23.5)	20 (15, 25)	0 (0, 1)	49.330	<0.001^**^	HR, MR>LR
suicidal ideation scores	N/A	7 (3, 8.5)	7 (5.25, 8.75)	0 (0, 1)	41.700	<0.001^**^	HR, MR>LR
Cytokines							
IL-6 (pg/ml)	0.73 (0.62, 1.34)	0.62 (0.42, 1.06)	0.54 (0.33, 0.77)	0.35 (0.30, 0.48)	20.082	<0.001^**^	HC>MR, LR HR>LR
TNF-α (pg/ml)	4.36 (3.81, 5.24)	2.93 (1.36, 4.10)	1.67 (1.16, 3.81)	1.37 (1.14, 1.69)	32.466	<0.001^**^	HC>HR, MR, LR HR>LR
IFN-γ (pg/ml)	14.84 (13.53, 19.36)	12.52 (4.36, 20.61)	6.72 (3.50, 13.66)	3.75 (2.81, 8.36)	22.157	<0.001^**^	HC>MR, LR HR>LR
IL-10 (pg/ml)	0.22 (0.18, 0.27)	0.27 (0.16, 0.37)	0.14 (0.09, 0.29)	0.12 (0.09, 0.18)	19.907	<0.001^**^	HC>MR, LR HR>MR, LR
IL-1β (pg/ml)	0.12 (0.08, 0.17)	0.06 (0.02, 0.17)	0.06 (0.01, 0.20)	0.03 (0.01, 0.10)	13.845	0.003^*^	HC>MR, LR

BMI, Body mass index; IL, interleukin; TNF, tumor necrosis factor; INF, Interferon; HC, health control.

HR, High suicide risk group; MR, Medium suicide risk group; LR, Low suicide risk group.

*p<0.05, **p<0.001.

P#: *post hoc* pairwise comparisons.

### 3.2 Inflammatory markers and suicide risk

#### 3.2.1 Group comparison of plasma cytokine concentrations

**Figure 1:**
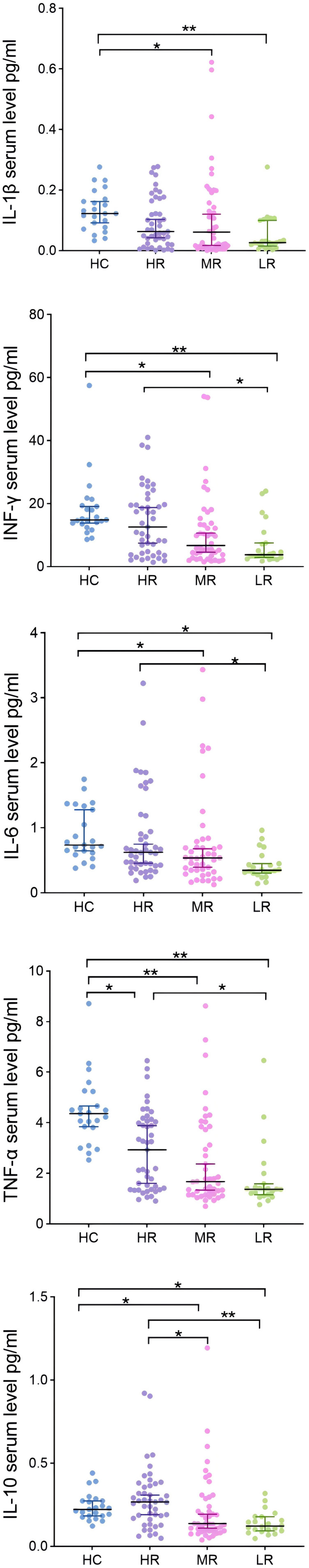
Pro-inflammatory factors and IL-10 levels in four groups. *p < 0.05, **p < 0.001.

Cytokine levels between groups are shown in **Figure 1**. Compared to the entire group of MDD patients, HCs showed significantly higher levels of four cytokines (**Table 2**), except IL-10(IL-10 did not show significant differences between HCs and patients). All five cytokines were at higher levels in the HC than in the LR and MR groups, and TNF-α levels were higher in the HC than in the HR group(t=3.296, p=0.006). Levels of four cytokines (except IL-1β) were significantly different among the three suicidal behavior subgroups [IL-6: H=10.281, p=0.006; TNF-α: H=8.960, p=0.011; INF-γ: H=9.125, p=0.010; IL-10: H=14.305 p=0.001]. Due to the mismatch in severity of depressive symptoms among patients in the three different suicide risk groups, we further controlled for HAMD-17 scores using ANCOVA. Differences in the levels of four cytokines remained significant among the three groups, with the following values: IL-6 [F(2, 108) = 4.615], p=0.012; TNF-α [F(2, 108) = 4.090], p=0.019; IFN-γ [F(2, 108) = 4.370], p=0.015 and IL-10 [F(2, 108) = 5.841], p=0.004. Pairwise comparisons showed that plasma concentrations of the four cytokines (except IL-1β) were higher in the HR than in the LR group (t=3.260, p=0.007 for IL-6; t=2.774, p=0.033 for TNF-α; t=3.004, p=0.016 for INF-γ; t=3.892, p=0.001 for IL-10), that there were no statistically significant differences between the MR and HR groups for the four cytokines (except IL-10), and that there was no statistically significant difference in the levels of all cytokines between the MR and LR groups. However, it is noteworthy that IL-10 level in the HR was significantly higher than in both the MR (t=2.821, p=0.028) and LR groups (t=3.892, p=0.001). All of the above pairwise comparisons were Bonferroni corrected.

**Table 2:** HCs VS MDD.

Cytokines	IL-6 (pg/ml)	TNF-α (pg/ml)	INF-γ (pg/ml)	IL-1β (pg/ml)	IL-10 (pg/ml)
HC	0.73 (0.62, 1.34)	4.36 (3.81, 5.24)	14.84 (13.53, 19.36)	0.12 (0.08, 0.17)	0.22 (0.18,0.27)
MDD	0.53 (0.34, 0.79)	1.70 (1.28, 3.85)	7.51 (3.42, 17.4)	0.05 (0.01,0.13)	0.18 (0.11,0.30)
Z	-3.074	-4.936	-3.532	-3.175	-1.699
p	0.002^*^	<0.001^**^	<0.001^**^	0.002^*^	0.089

*p<0.05, **p<0.001.

#### 3.2.2 The associations between cytokine levels with depressive symptoms and different dimensions of suicidality

**Figure 2:**
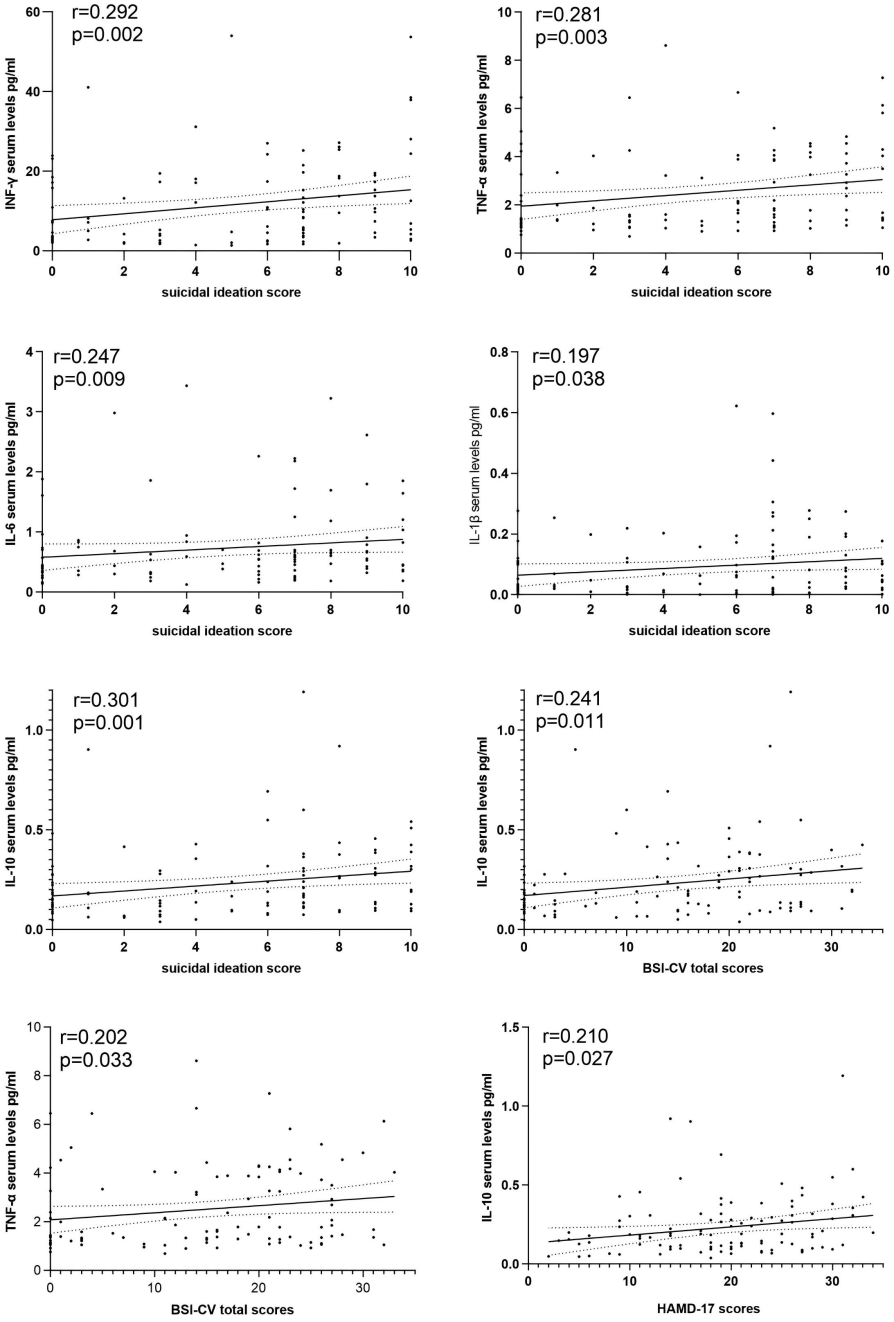
The associations between cytokine levels with depressive symptoms and different dimensions of suicidality in patients.

Spearman correlation analysis showed that IL-10 correlated positively with HAMD-17 total score (r=0.210, p=0.027), BSI-CV total score (r=0.241, p=0.011), and suicidal ideation score (r=0.301, p=0.001); TNF-α correlated positively with BSI-CV total score (r=0.202, p=0.033). Furthermore, IL-6, TNF-α, IFN-γ, and IL-1β all showed some correlation with suicidal ideation scores (all p<0.05), as shown in **Table 3** and **Figure 2**. In addition, IL-10 and IL-6, IL-1β, TNFα, and IFN-γ were all significantly positively correlated with each other (r=0.631, 0.489, 0.654, and 0.736, all p<0.001).

**Table 3:** The associations between cytokine levels with depressive symptoms and different dimensions of suicidality in patients.

Variables	IL-6		TNF-α		INF-γ		IL-10		IL-Iβ	
	r	p	r	P	r	p	r	p	r	P
HAMD-17 scores	0.062	0.515	0.132	0.167	0.041	0.666	0.210	0.027^*^	0.099	0.301
BSI-CV total scores	0.177	0.063	0.202	0.033^*^	0.170	0.075	0.241	0.011^*^	0.178	0.068
suicidal ideation scores	0.247	0.009^*^	0.281	0.003^*^	0.292	0.002^*^	0.301	0.001^*^	0.197	0.038^*^

*p<0.05.

#### 3.2.3 Independent influencing factors of the total scores on the HAMD-17 and BSI-CV

With HAMD-17 and BSI-CV total scores as the dependent variables, and gender, age, BMI, duration of illness, and inflammatory cytokines as independent variables included in a multivariate linear regression model, IL-10 significantly affected both the HAMD-17 (β=0.285, t=3.041, p=0.003) and the BSI-CV total scores (β=0.270, t=2.897, p=0.005); age significantly influenced HAMD-17 total score (β=-0.207, t=-2.181, p=0.031), and there was a significant difference by gender (with females scoring higher than males) on the BSI-CV total score (β=0.201, t=2.199, p=0.030). See **Table 4** for details.

**Table 4:** Independent correlation of the total scores on the HAMD-17 and BSI-CV.

Variables	Beta		t		P	
	HAMD-17	BSI-CV	HAMD-17	BSI-CV	HAMD-17	BSI-CV
IL-10	0.285	0.270	3.041	2.897	0.003	0.005
BMI	-0.078	0.025	-0.848	0.276	0.398	0.783
Gender (1)	0.109	0.201	1.187	2.199	0.238	0.030
Disease Duration	0.106	0.068	1.131	0.734	0.261	0.464
Age	-0.207	-0.163	-2.181	-1.727	0.031	0.087

#### 3.2.4 Independent predictors of suicidal behavior in patients with depressive disorder

IL-10 level in the HR was significantly higher than that in the MR (p=0.028) and LR groups (p=0.001) in each suicidal behavior subgroups, and IL-10 significantly affected total BSI-CV score. Furthermore, the univariate regression model with IL-10 as the independent variable and history of suicide attempts or not as the dependent variable was significant (OR=2.684, 95% CI 1.455-4.951 p=0.002). Considering our small sample size, we controlled for gender, age, BMI, duration of illness, and severity of depressive symptoms in the same model. IL-10 remained independently associated with suicidal behavior in depressed patients (OR=3.224, 95% CI 1.571-6.619 p=0.001).

#### 3.2.5 Prediction of the diagnostic efficacy of IL-10 for suicide behavior in patients with depression

**Figure 3:**
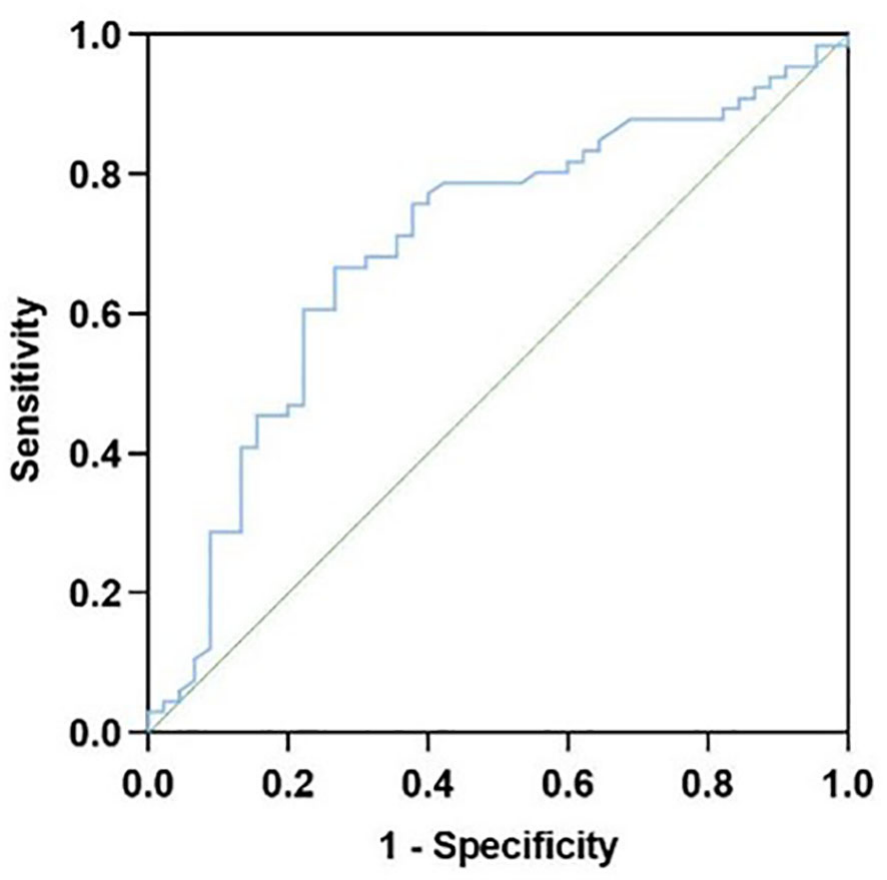
Receiver operating characteristic (ROC) curve for IL-10.

The presence or absence of suicidal behavior was used as the status variable in patients with depression, and IL-10 was employed as the test variable for the ROC curve analysis. The results suggested that IL-10 has a certain diagnostic value, with an AUC area of 0.696 (0.594, 0.798) (p < 0.001), a specificity of 0.667, a sensitivity of 0.733, a Youden’s index of 0.400, and the optimal cutoff value at 0.182 pg/ml, as shown in **Figure 3**.

## 4 Discussion

In this study, we investigated the relationship between plasma cytokine levels and suicide risk stratification in adolescents with depression, and found high levels of IL-6, TNFα, IFN-γ, and IL-10 in the high-suicide-risk group, which is consistent with the results of previous studies (12, 43–45). Similar results were obtained in ANCOVA after controlling for the severity of depressive symptoms. In a subsequent two-by-two comparison, IL-10 in particular, was significantly higher than in the low and intermediate risk groups. As previously found in adults with depression (46–49), the levels of all five cytokines were positively correlated with intensity of suicidal ideation in adolescents with depression. Unexpectedly, there was a lack of association between the levels of these four pro-inflammatory factors and depressive symptoms. Also unexpected was the fact that all five cytokines were at higher levels in the healthy control group than in the depressed group. In most studies these cytokines were shown to be at higher levels in adolescent depressed patients (50–53). Binary logistic regression analysis also showed that IL-10 levels still independently influenced suicidal behavior in depressed patients when controlling for severity of depressive symptoms, and IL-10 was of value in the diagnosis of suicide attempt status by the ROC curve test. Thus, the main finding of this study is that higher levels of serum cytokines may be associated with suicidal ideation/behavior in adolescents with depression, and this association does not appear to be influenced by depression severity.

Immune dysfunction may be one of the important factors contributing to the development of suicidal symptoms, and our findings are to some extent consistent with previous reports showing that plasma levels of inflammatory cytokines were higher in adolescents with depression who had a history of suicidal behavior than in those who did not have suicidal ideation. A previous review reported a cross-sectional association between elevated inflammatory markers in cerebrospinal fluid and serum, including IFN-γ, TNF-α, IL-6, and IL-1β, and suicidal behavior in children and adolescents (54). A cohort study of depression and anxiety in child adolescents by Amitai et al. also found that changes in IL-6 before and after treatment was the strongest predictor in a logistic regression model predicting the risk of fluoxetine-associated suicide. That is, high post-treatment levels of IL-6 can predict fluoxetine-associated suicidal behaviors to some extent (55). Additionally, Pandey examined the levels of immune markers in the cerebrospinal fluid of 24 adolescents who died by suicide, and higher levels of mRNA and protein expression of IL-6, TNFα, and IL-1β were detected in the prefrontal cortex compared to healthy controls (56), although only 33.3% (8) of these victims had been diagnosed with depression during their lifetime, suggesting that inflammatory cytokines association with adolescent suicidal ideation is not dependent on depression.

In our study, depressive symptoms were not substantially related with these four pro-inflammatory cytokines levels, and even healthy controls showed higher levels of inflammatory factors than the depressed group. Some previous studies did find lower cytokine levels associated with depression. In a 2020 case-control study, serum levels of IFN-γ were significantly lower in patients with MDD than in controls and were negatively correlated with depressive symptoms (57). In a small-sample prospective study, Lee et al. found that compared to healthy peers, adolescents with first-onset major depression possessed lower IL-2, IFN-γ and TNF-α concentrations prior to their first treatment with antidepressant medication (58). Ovaskaine et al. also observed in a large study based on a middle-aged population that men with depressive symptoms exhibited lower levels of IL-1β levels (59). Additionally, a Korean study found lower levels of IFN-γ, IL-2, and IL-4 in adult depressed patients compared to healthy controls. In the same study they also found gender-based differences in cytokine levels, that is, male patients had higher plasma levels of IFN-γ, TNF-α, and IL-6 than female patients (60). Admittedly, like our study, the proportion of females in these studies was significantly higher than that of males, therefore cytokine levels may have been influenced by female sex hormones, leading to the heterogeneity of results. Additionally, Buspavanich and his team found that both the speed of onset of depression and the duration of depressive episodes affected cytokine levels in the body (61). We also speculate that differences in the age of recruited subjects, childhood traumatic experiences, effects of medications taken, stage of depressive episodes (acute, remission), presence or absence of psychotic symptoms, as well as differences in sample size and testing methods also led to the variation in results. Simultaneously, the contradictory results reflect the complexity and diversity of mechanisms involved in depression.

We observed that only one inflammatory marker, IL-10, was elevated in patients with suicidal ideation and behavior compared to adolescent depressed patients with suicidal ideation but no suicidal behavior in this study. Moreover, IL-10 correlated positively with severity of depressive symptoms, intensity of suicidal ideation, and suicide risk, and was an independent risk factor for suicidal behaviors and depressive symptoms in adolescent depressed patients in subsequent regression analyses. Therefore, we hypothesized that IL-10 may be a potential biomarker for predicting suicidal behavior. IL-10 is a homodimer of 178 amino acids with multiple functions, mainly secreted from monocyte macrophages, T cells, and B cells *in vivo* (62), and plays an immunosuppressive or immunostimulatory role in different cellular responses (63). Previously, IL-10 has been widely studied in the pathogenesis of depression, although the relationship between it and depression is unclear, and many studies even present opposite conclusions. Research has noted that these divergent findings may be attributed to the regular changes in IL-10 levels as the disease progresses (64). However, studies examining both IL-10 and suicide are scarce. Therefore, the focus of this study was more on the potential involvement of IL-10 in suicide. Previously O’Donovan found that IL-10 levels in depressed patients with high levels of suicidal ideation were significantly higher than those low in suicidal ideation, and the difference in inflammatory factors remained statistically significant when controlling for the severity of depressive symptoms (12), suggesting that suicidal ideation is independently correlated with elevated IL-10 levels. Additionally, in his study, IL-6 was elevated along with IL-10, which is consistent with our study, where we found that IL-6 and IL-10 were positively and significantly correlated. It has been suggested that there may be some kind of feed-forward regulation in depression, with IL-6 driving IL-10 release and thereby suppressing the inflammatory response (64). We hypothesize that this regulatory mechanism may also be present in the inflammatory response due to suicidal ideation, which could explain to some extent the differences in IL-10 in the medium- to high-suicide risk groups, rather than the IL-6. Additionally, similar to our findings, some reviews revealed that IL-10 is also elevated along with pro-inflammatory cytokines such as IL-1β, TNF-α, and others in many other pathophysiological situations, although the pathway that induces IL-10 expression negatively regulates the synthesis of these pro-inflammatory factors (62).

Increasing evidence indicates that aberrant activation of the kynurenine pathway (KP) may be one of the underlying neurobiological mechanisms of suicide, which mainly manifests by decreased kynurenine acid (KynA) synthesis and increased synthesis of neurotoxic metabolites (65). KynA has neuroprotective effects, mainly in terms of its ability to reduce the neurotoxicity of glutamate and enhance inter-synaptic plasticity (66). KP-related neurotoxic metabolites mainly refer to 3-hydroxykynurenine and quinolinic acid, which can lead to neurotoxicity through stimulation of neuronal apoptosis, enhancement of oxidative stress, reduction of brain-derived neurotrophic factor production, and stimulation of increased glutamate release (66–68). Imbalances in this series of neurotransmitter and neuroinflammatory metabolites may be among the driving factors leading to the emergence of suicidal ideation/behavior. Previous studies have used elevated kynurenine/tryptophan (KYN/TRP) ratios as a marker of KP dysregulation and found increased KYN/TRP in individuals with suicide attempts (69–71). Nettis et al. observed that elevated KYN/TRP was significantly associated with the elevation of IL-10 in individuals with suicidal ideation (72). IL-10 primarily functions as an anti-inflammatory factor and we hypothesize that elevation of IL-10 may be in response to aberrant activation of KP and thus maintain the homeostasis of the neuroinflammatory environment.

Our study has certain limitations. First, as a cross-sectional study, it could not reveal the causal directionality between changes in peripheral inflammatory factors and suicide. Second, this study is limited by a small sample size, poor representation of the general population, as well as limited statistical power. Third, the effect of antidepressants on cytokine levels could not be completely excluded despite that we recruited patients who had discontinued antidepressants for at least 2 weeks. Fourth, observation of suicide in this study was rather one-sided, and more dimensions of suicide need to be assessed in the future (including frequency of suicide, methods of suicide, and severity of suicidal behavior). Fifth, possibly due to a flaw in the assay methodology, very few subjects had plasma levels of cytokines below the threshold that could be detected and could not be included in our statistical analysis. Sixth, we did not follow up the included patients longitudinally to rule out changes in the diagnosis of depression, therefore longitudinal observations are necessary in future studies.

## 5 Conclusion

In summary, our study has the following the clinical significance: We found that high levels of inflammatory factors are closely associated with risk of suicide in adolescent depressed patients and appear to be unaffected by depressive symptoms. In particular, IL-10 may be a promising biomarker for predicting suicidal behaviors in adolescent depressed patients, especially for assessing the risk of suicidal behaviors in patients with clear suicidal ideation, and for intervening in advance of the development of suicidal behavior. It also suggests the role of anti-inflammatory factors in the mechanism of suicide, and more studies are required to explore the relationship between anti-inflammatory factors such as IL-1 receptor antagonist (IL-1ra), IL-4, IL-11, IL-10, and suicide.

## Data Availability

The raw data supporting the conclusions of this article will be made available by the authors, without undue reservation.

## References

[1] Fazel S Runeson B. Suicide. New Engl J Med. (2020) 382:266–74. doi: 10.1056/NEJMra1902944 PMC7116087 31940700

[2] Copeland WE Goldston DB Costello EJ. Adult associations of childhood suicidal thoughts and behaviors: A prospective, longitudinal analysis. J Am Acad Child Adolesc Psychiatry. (2017) 56:958–965.e954. doi: 10.1016/j.jaac.2017.08.015 29096778 PMC6501553

[3] Bachmann S. Epidemiology of suicide and the psychiatric perspective. Int J Environ Res Public Health. (2018) 15:1425. doi: 10.3390/ijerph15071425 29986446 PMC6068947

[4] Lew B Huen J Yu P Yuan L Wang DF Ping F. Associations between depression, anxiety, stress, hopelessness, subjective well-being, coping styles and suicide in Chinese university students. PloS One. (2019) 14:e0217372. doi: 10.1371/journal.pone.0217372 31260454 PMC6602174

[5] Lewis CP Camsari DD Sonmez AI Nandakumar AL Gresbrink MA Daskalakis ZJ. Preliminary evidence of an association between increased cortical inhibition and reduced suicidal ideation in adolescents treated for major depression. J Affect Disord. (2019) 244:21–4. doi: 10.1016/j.jad.2018.09.079 PMC6231405 30292987

[6] Liu D Liu S Deng H Qiu L Xia B Liu W. Depression and suicide attempts in Chinese adolescents with mood disorders: the mediating role of rumination. Eur Arch Psychiatry Clin Neurosci. (2023) 273:931–40. doi: 10.1007/s00406-022-01444-2 35763221

[7] Courtet P Jaussent I Genty C Dupuy AM Guillaume S Ducasse D. Increased CRP levels may be a trait marker of suicidal attempt. Eur Neuropsychopharmacol: J Eur Coll Neuropsychopharmacol. (2015) 25:1824–31. doi: 10.1016/j.euroneuro.2015.05.003 26032768

[8] González-Castro TB Tovilla-Zárate CA López-Narváez ML Genis-Mendoza AD Juárez-Rojop IE. Interleukin-6 levels in serum, plasma, and cerebral spinal fluid in individuals with suicide behavior: systematic review and meta-analysis with meta-regression. J Interferon Cytokine Res: Off J Int Soc Interferon Cytokine Res. (2021) 41:258–67. doi: 10.1089/jir.2020.0265 34280025

[9] Kappelmann N Arloth J Georgakis MK Czamara D Rost N Ligthart S. Dissecting the association between inflammation, metabolic dysregulation, and specific depressive symptoms: A genetic correlation and 2-sample mendelian randomization study. JAMA Psychiatry. (2021) 78:161–70. doi: 10.1001/jamapsychiatry.2020.3436 PMC7577200 33079133

[10] Köhler CA Freitas TH Maes M de Andrade NQ Liu CS Fernandes BS. Peripheral cytokine and chemokine alterations in depression: a meta-analysis of 82 studies. Acta Psychiatrica Scandinavica. (2017) 135:373–87. doi: 10.1111/acps.12698 28122130

[11] Karlović D Serretti A Vrkić N Martinac M Marčinko D. Serum concentrations of CRP, IL-6, TNF-α and cortisol in major depressive disorder with melancholic or atypical features. Psychiatry Res. (2012) 198:74–80. doi: 10.1016/j.psychres.2011.12.007 22386567

[12] O’Donovan A Rush G Hoatam G Hughes BM McCrohan A Kelleher C. Suicidal ideation is associated with elevated inflammation in patients with major depressive disorder. Depress Anxiety. (2013) 30:307–14. doi: 10.1002/da.2013.30.issue-4 23504697

[13] Beurel E Toups M Nemeroff CB. The bidirectional relationship of depression and inflammation: double trouble. Neuron. (2020) 107:234–56. doi: 10.1016/j.neuron.2020.06.002 PMC7381373 32553197

[14] Rush G O’Donovan A Nagle L Conway C McCrohan A O’Farrelly C. Alteration of immune markers in a group of melancholic depressed patients and their response to electroconvulsive therapy. J Affect Disord. (2016) 205:60–8. doi: 10.1016/j.jad.2016.06.035 PMC5291160 27414954

[15] Moughrabi S Evangelista LS Habib SI Kassabian L Breen EC Nyamathi A. In patients with stable heart failure, soluble TNF-receptor 2 is associated with increased risk for depressive symptoms. Biol Res Nurs. (2014) 16:295–302. doi: 10.1177/1099800413496454 23904128 PMC4447109

[16] Guerrero LR Hong S Tarraf W Perreira K Camacho Á Kohn JN. Association of anxiety and depressive symptoms with C-reactive protein in diverse Latinos: Results from the Hispanic Community Health Study/Study of Latinos (HCHS/SOL). PloS One. (2023) 18:e0289833. doi: 10.1371/journal.pone.0289833 37594961 PMC10437793

[17] von Känel R Mausbach BT Mills PJ Dimsdale JE Patterson TL Ancoli-Israel S. Longitudinal relationship of low leisure satisfaction but not depressive symptoms with systemic low-grade inflammation in dementia caregivers. J Gerontol Ser B psychol Sci Soc Sci. (2014) 69:397–407. doi: 10.1093/geronb/gbt020 23650246 PMC3983912

[18] Fragoso YD Frota ER Lopes JS Noal JS Giacomo MC Gomes S. Severe depression, suicide attempts, and ideation during the use of interferon beta by patients with multiple sclerosis. Clin Neuropharmacol. (2010) 33:312–6. doi: 10.1097/WNF.0b013e3181f8d513 21079457

[19] Dieperink E Ho SB Tetrick L Thuras P Dua K Willenbring ML. Suicidal ideation during interferon-alpha2b and ribavirin treatment of patients with chronic hepatitis C. Gen Hosp Psychiatry. (2004) 26:237–40. doi: 10.1016/j.genhosppsych.2004.01.003 15121353

[20] Dickerson SS Gable SL Irwin MR Aziz N Kemeny ME. Social-evaluative threat and proinflammatory cytokine regulation: an experimental laboratory investigation. psychol Sci. (2009) 20:1237–44. doi: 10.1111/j.1467-9280.2009.02437.x PMC2761517 19754527

[21] Perna L Trares K Perneczky R Tato M Stocker H Möllers T. Risk of late-onset depression and cognitive decline: results from inflammatory proteome analyses in a prospective population-based cohort study. Am J Geriatric Psychiatry: Off J Am Assoc Geriatric Psychiatry. (2022) 30:689–700. doi: 10.1016/j.jagp.2021.12.001 34961662

[22] Ryan KM McLoughlin DM. Peripheral blood inflammatory markers in depression: Response to electroconvulsive therapy and relationship with cognitive performance. Psychiatry Res. (2022) 315:114725. doi: 10.1016/j.psychres.2022.114725 35870295

[23] Nelson AM Erdmann AA Coe CL Juckett MB Morris K Knight JM. Inflammatory cytokines and depression symptoms following hematopoietic cell transplantation. Brain Behav Immun. (2023) 112:11–7. doi: 10.1016/j.bbi.2023.05.012 PMC10524437 37236325

[24] Isung J Aeinehband S Mobarrez F Nordström P Runeson B Asberg M. High interleukin-6 and impulsivity: determining the role of endophenotypes in attempted suicide. Trans Psychiatry. (2014) 4:e470. doi: 10.1038/tp.2014.113 PMC4350519 25335166

[25] Guo Y Jiang X Jia L Zhu Y Han X Wu Y. Wang D et al: Altered gray matter volumes and plasma IL-6 level in major depressive disorder patients with suicidal ideation. NeuroImage Clin. (2023) 38:103403. doi: 10.1016/j.nicl.2023.103403 37079937 PMC10148078

[26] Fernández-Sevillano J González-Ortega I MacDowell K Zorrilla I López MP Courtet P. Inflammation biomarkers in suicide attempts and their relation to abuse, global functioning and cognition. World J Biol Psychiatry: Off J World Fed Societies Biol Psychiatry. (2022) 23:307–17. doi: 10.1080/15622975.2021.1988703 34730074

[27] Bramness JG Pandey S Moe JS Toft H Lien L Walby FA. Levels of IL-6 are associated with lifetime attempted suicide in alcohol use disorder patients. Neuropsychiatr Dis Treat. (2023) 19:2141–8. doi: 10.2147/NDT.S413101 PMC10578180 37849526

[28] Dolsen EA Prather AA Lamers F Penninx B. Suicidal ideation and suicide attempts: associations with sleep duration, insomnia, and inflammation. psychol Med. (2021) 51:2094–103. doi: 10.1017/S0033291720000860 32321599

[29] Bastos CR Gazal M Quevedo LA Costa JL Wiener CD Jansen K. Polymorphism in CRHR1 gene affects the IL-1β levels in suicidal attempters. J Psychiatr Res. (2017) 86:34–8. doi: 10.1016/j.jpsychires.2016.11.009 27894002

[30] Coryell W Wilcox H Evans SJ Pandey GN Jones-Brando L Dickerson F. Aggression, impulsivity and inflammatory markers as risk factors for suicidal behavior. J Psychiatr Res. (2018) 106:38–42. doi: 10.1016/j.jpsychires.2018.09.004 30261413

[31] Lee JY Jhon M Kim JW Kang HJ Kim SW Shin IS. Interaction effect between childhood abuse and interleukin-1β levels on suicidality in depressed patients. Eur Arch Psychiatry Clin Neurosci. (2022) 272:1535–46. doi: 10.1007/s00406-022-01408-6 35467148

[32] Monfrim X Gazal M De Leon PB Quevedo L Souza LD Jansen K. Immune dysfunction in bipolar disorder and suicide risk: is there an association between peripheral corticotropin-releasing hormone and interleukin-1β? Bipolar Disord. (2014) 16:741–7. doi: 10.1111/bdi.2014.16.issue-7 24862833

[33] Lu J Li S Li H Mou T Zhou L Huang B. Changes in plasma NPY, IL-1β And hypocretin in people who died by suicide. Neuropsychiatr Dis Treat. (2019) 15:2893–900. doi: 10.2147/NDT.S219962 PMC6791488 31632037

[34] Kim YK Hong JP Hwang JA Lee HJ Yoon HK Lee BH. TNF-alpha -308G>A polymorphism is associated with suicide attempts in major depressive disorder. J Affect Disord. (2013) 150:668–72. doi: 10.1016/j.jad.2013.03.019 23608119

[35] Zhai S Qu Y Zhang D Li T Xie Y Wu X. Depressive symptoms predict longitudinal changes of chronic inflammation at the transition to adulthood. Front Immunol. (2022) 13:1036739. doi: 10.3389/fimmu.2022.1036739 36685498 PMC9846044

[36] de Medeiros Alves V ES ACP de Souza EVM de Lima Francisco LCF de Moura EL de-Melo-Neto VL. Suicide attempt in mental disorders (MeDi): Association with 5-HTT, IL-10 and TNF-alpha polymorphisms. J Psychiatr Res. (2017) 91:36–46. doi: 10.1016/j.jpsychires.2017.02.022 28314127

[37] Miná VA Lacerda-Pinheiro SF Maia LC Pinheiro RF Jr. Meireles CB de Souza SI. The influence of inflammatory cytokines in physiopathology of suicidal behavior. J Affect Disord. (2015) 172:219–30. doi: 10.1016/j.jad.2014.09.057 25451421

[38] Lang X Trihn TH Wu HE Tong Y Xiu M Zhang XY. Association between TNF-alpha polymorphism and the age of first suicide attempt in chronic patients with schizophrenia. Aging. (2020) 12:1433–45. doi: 10.18632/aging.102692 PMC7053594 31954374

[39] Mendlovic S Mozes E Eilat E Doron A Lereya J Zakuth V. Immune activation in non-treated suicidal major depression. Immunol Lett. (1999) 67:105–8. doi: 10.1016/S0165-2478(98)00145-X 10232390

[40] Ganança L Oquendo MA Tyrka AR Cisneros-Trujillo S Mann JJ Sublette ME. The role of cytokines in the pathophysiology of suicidal behavior. Psychoneuroendocrinology. (2016) 63:296–310. doi: 10.1016/j.psyneuen.2015.10.008 26546783 PMC4910882

[41] Omrani MD Bushehri B Bagheri M Salari-Lak S Alipour A Anoshae MR. Role of IL-10 -1082, IFN-gamma +874, and TNF-alpha -308 genes polymorphisms in suicidal behavior. Arch Suicide Res: Off J Int Acad Suicide Res. (2009) 13:330–9. doi: 10.1080/13811110903266418 19813110

[42] Gabbay V Klein RG Guttman LE Babb JS Alonso CM Nishawala M. A preliminary study of cytokines in suicidal and nonsuicidal adolescents with major depression. J Child Adolesc Psychopharmacol. (2009) 19:423–30. doi: 10.1089/cap.2008.0140 PMC2778037 19702494

[43] Lozupone M Donghia R Sardone R Mollica A Berardino G Lampignano L. Apolipoprotein E genotype, inflammatory biomarkers, and non-psychiatric multimorbidity contribute to the suicidal ideation phenotype in older age. Salus Apulia Study J Affect Disord. (2022) 319:202–12. doi: 10.1016/j.jad.2022.09.046 36155237

[44] Wiebenga JXM Heering HD Eikelenboom M van Hemert AM van Oppen P Penninx B. Associations of three major physiological stress systems with suicidal ideation and suicide attempts in patients with a depressive and/or anxiety disorder. Brain Behav Immun. (2022) 102:195–205. doi: 10.1016/j.bbi.2022.02.021 35202735

[45] Ninla-Aesong P Puangsri P Kietdumrongwong P Jongkrijak H Noipha K. Being overweight and obese increases suicide risk, the severity of depression, and the inflammatory response in adolescents with major depressive disorders. Front Immunol. (2023) 14:1197775. doi: 10.3389/fimmu.2023.1197775 38022570 PMC10646409

[46] Thomas N Armstrong CW Hudaib AR Kulkarni J Gurvich C. A network meta-analysis of stress mediators in suicide behaviour. Front Neuroendocrinol. (2021) 63:100946. doi: 10.1016/j.yfrne.2021.100946 34481858

[47] Kim JM Kim JW Kang HJ Choi W Lee JY Kim SW. Predicting suicidal ideation using multiple serum biomarkers in patients with acute coronary syndrome. J Affect Disord. (2024) 351:915–9. doi: 10.1016/j.jad.2024.02.008 38342323

[48] Zeng Y Li W Chen X You Z Mai S Lan X. Mediating effect of inflammation on the relationship between sleep disruption and suicidal ideation in major depressive disorder. J Affect Disord. (2024) 352:371–8. doi: 10.1016/j.jad.2024.02.078 38401806

[49] Defayette AB Esposito-Smythers C Cero I Kleiman EM López R Jr. Harris KM. Examination of proinflammatory activity as a moderator of the relation between momentary interpersonal stress and suicidal ideation. Suicide Life-threatening Behav. (2023) 53:922–39. doi: 10.1111/sltb.12993 PMC10840613 37578098

[50] Ho TC Kulla A Teresi GI Sisk LM Rosenberg-Hasson Y Maecker HT. Inflammatory cytokines and callosal white matter microstructure in adolescents. Brain Behav Immun. (2022) 100:321–31. doi: 10.1016/j.bbi.2021.12.003 PMC11849988 34896593

[51] Ferencova N Visnovcova Z Ondrejka I Funakova D Hrtanek I Kelcikova S. Evaluation of inflammatory response system (IRS) and compensatory immune response system (CIRS) in adolescent major depression. J Inflammation Res. (2022) 15:5959–76. doi: 10.2147/JIR.S387588 PMC9596279 36303711

[52] Liu L Yang X Yang C Tian Y Li W Xia L. Associations between insomnia symptoms and inflammatory cytokines in adolescents with first-episode and recurrent major depressive disorder. J Affect Disord. (2024) 350:110–7. doi: 10.1016/j.jad.2024.01.031 38220096

[53] Li Y Jinxiang T Shu Y Yadong P Ying L Meng Y. Childhood trauma and the plasma levels of IL-6, TNF-α are risk factors for major depressive disorder and schizophrenia in adolescents: A cross-sectional and case-control study. J Affect Disord. (2022) 305:227–32. doi: 10.1016/j.jad.2022.02.020 35151670

[54] Kim JW Szigethy EM Melhem NM Saghafi EM Brent DA. Inflammatory markers and the pathogenesis of pediatric depression and suicide: a systematic review of the literature. J Clin Psychiatry. (2014) 75:1242–53. doi: 10.4088/JCP.13r08898 25470085

[55] Amitai M Taler M Ben-Baruch R Lebow M Rotkopf R Apter A. Increased circulatory IL-6 during 8-week fluoxetine treatment is a risk factor for suicidal behaviors in youth. Brain Behav Immun. (2020) 87:301–8. doi: 10.1016/j.bbi.2019.12.017 31887416

[56] Pandey GN. Inflammatory and innate immune markers of neuroprogression in depressed and teenage suicide brain. Mod Trends Pharmacopsychiatry. (2017) 31:79–95. doi: 10.1159/000470809 28738369

[57] Daria S Proma MA Shahriar M Islam SMA Bhuiyan MA Islam MR. Serum interferon-gamma level is associated with drug-naïve major depressive disorder. SAGE Open Med. (2020) 8:2050312120974169. doi: 10.1177/2050312120974169 33282305 PMC7682211

[58] Lee H Song M Lee J Kim JB Lee MS. Prospective study on cytokine levels in medication-naïve adolescents with first-episode major depressive disorder. J Affect Disord. (2020) 266:57–62. doi: 10.1016/j.jad.2020.01.125 32056928

[59] Ovaskainen Y Koponen H Jokelainen J Keinänen-Kiukaanniemi S Kumpusalo E Vanhala M. Depressive symptomatology is associated with decreased interleukin-1 beta and increased interleukin-1 receptor antagonist levels in males. Psychiatry Res. (2009) 167:73–9. doi: 10.1016/j.psychres.2007.12.004 19346005

[60] Kim YK Na KS Shin KH Jung HY Choi SH Kim JB. Cytokine imbalance in the pathophysiology of major depressive disorder. Prog Neuropsychopharmacol Biol Psychiatry. (2007) 31:1044–53. doi: 10.1016/j.pnpbp.2007.03.004 17433516

[61] Buspavanich P Adli M Himmerich H Berger M Busche M Schlattmann P. Faster speed of onset of the depressive episode is associated with lower cytokine serum levels (IL-2, -4, -6, -10, TNF-α and IFN-γ) in patients with major depression. J Psychiatr Res. (2021) 141:287–92. doi: 10.1016/j.jpsychires.2021.06.033 34271459

[62] Saraiva M O’Garra A. The regulation of IL-10 production by immune cells. Nat Rev Immunol. (2010) 10:170–81. doi: 10.1038/nri2711 20154735

[63] Nagata K Nishiyama C. IL-10 in mast cell-mediated immune responses: anti-inflammatory and proinflammatory roles. Int J Mol Sci. (2021) 22:4972. doi: 10.3390/ijms22094972 34067047 PMC8124430

[64] Wiener CD Moreira FP Portela LV Strogulski NR Lara DR da Silva RA. Interleukin-6 and Interleukin-10 in mood disorders: A population-based study. Psychiatry Res. (2019) 273:685–9. doi: 10.1016/j.psychres.2019.01.100 31207853

[65] Bryleva EY Brundin L. Kynurenine pathway metabolites and suicidality. Neuropharmacology. (2017) 112:324–30. doi: 10.1016/j.neuropharm.2016.01.034 PMC5998805 26820800

[66] Kopra E Mondelli V Pariante C Nikkheslat N. Ketamine’s effect on inflammation and kynurenine pathway in depression: A systematic review. J Psychopharmacol (Oxford England). (2021) 35:934–45. doi: 10.1177/02698811211026426 PMC8358579 34180293

[67] Bryleva EY Brundin L. Suicidality and activation of the kynurenine pathway of tryptophan metabolism. Curr Topics Behav Neurosci. (2017) 31:269–84. doi: 10.1007/7854_2016_5 27221623

[68] Goda K Kishimoto R Shimizu S Hamane Y Ueda M. Quinolinic acid and active oxygens. Possible contribution of active Oxygens during cell death in the brain. Adv Exp Med Biol. (1996) 398:247–54. 8906272

[69] Sublette ME Galfalvy HC Fuchs D Lapidus M Grunebaum MF Oquendo MA. Plasma kynurenine levels are elevated in suicide attempters with major depressive disorder. Brain Behav Immun. (2011) 25:1272–8. doi: 10.1016/j.bbi.2011.05.002 PMC3468945 21605657

[70] Messaoud A Mensi R Douki W Neffati F Najjar MF Gobbi G. Reduced peripheral availability of tryptophan and increased activation of the kynurenine pathway and cortisol correlate with major depression and suicide. World J Biol Psychiatry: Off J World Fed Societies Biol Psychiatry. (2019) 20:703–11. doi: 10.1080/15622975.2018.1468031 29683396

[71] Bradley KA Case JA Khan O Ricart T Hanna A Alonso CM. The role of the kynurenine pathway in suicidality in adolescent major depressive disorder. Psychiatry Res. (2015) 227:206–12. doi: 10.1016/j.psychres.2015.03.031 PMC4430385 25865484

[72] Nettis MA Lombardo G Hastings C Zajkowska Z Mariani N Nikkheslat N. The interaction between kynurenine pathway, suicidal ideation and augmentation therapy with minocycline in patients with treatment-resistant depression. J Psychopharmacol (Oxford England). (2023) 37:531–8. doi: 10.1177/02698811231173588 PMC10291376 37183855

